# Non‐canonical function of transferrin receptor‐1 promotes breast cancer metastasis by activating HCK‒STAT3‒MMP9 signalling

**DOI:** 10.1002/ctm2.70731

**Published:** 2026-07-12

**Authors:** Qing Zhao, Yafang Wang, Pengfei Wang, Yaqi Ding, Rong Wang, Yanyan Shen, Biyu Yang, Yanfen Fang, Jian Ding, Yi Chen

**Affiliations:** ^1^ Nanchang University School of Pharmacy Nanchang University Nanchang PR China; ^2^ State Key Laboratory of Drug Research Division of Antitumor Pharmacology Shanghai Institute of Materia Medica, Chinese Academy of Sciences Shanghai PR China; ^3^ State Key Laboratory of Chemical Biology Division of Antitumor Pharmacology Shanghai Institute of Materia Medica, Chinese Academy of Sciences Shanghai PR China; ^4^ State Key Laboratory of Systems Medicine for Cancer and International Collaboration Cancer Center Shanghai Cancer Institute, Renji Hospital, Shanghai Jiao Tong University School of Medicine Shanghai PR China; ^5^ Shandong Laboratory of Yantai Drug Discovery Bohai Rim Advanced Research Institute for Drug Discovery Yantai PR China

**Keywords:** breast cancer, HCK, metastasis, STAT3, TfR‐1, USP32

## Abstract

**Background:**

Transferrin receptor 1 (TfR‐1), a key mediator of cellular iron uptake, is significantly upregulated across a broad spectrum of malignancies, and its overexpression highly correlated with poor clinical outcomes, such as in breast cancer. However, its precise role in breast cancer progression remains unclear.

**Methods:**

We integrated clinical breast cancer tissue specimens and public transcriptomic datasets to analyse the clinical correlation between TfR‐1 expression and tumour metastasis. A series of in vitro cell functional assays and in vivo xenograft tumour metastasis models were performed to validate the regulatory effect of TfR‐1 on breast cancer proliferation and metastasis. Co‑immunoprecipitation, phosphorylation detection and protein stability assays were further applied to dissect the intermolecular regulatory network among TfR‐1, Hematopoietic Cell Kinase (HCK) and Ubiquitin Specific Peptidase 32 (USP32).

**Results:**

Clinical data analysis revealed that elevated TfR‐1 expression was tightly linked to metastatic phenotype in breast cancer. Functional experiments confirmed that upregulated TfR‐1 robustly boosted the proliferative and metastatic capacity of breast cancer cells in vitro and in vivo. Mechanistically, TfR‐1 dual‑regulates HCK, it directly induces HCK phosphorylation, while simultaneously activating USP32 to block HCK protein degradation and sustain HCK abundance. Activated HCK further triggers STAT3 transcription factor signalling, which drives the upregulation and secretion of matrix metalloproteinase 9 (MMP9). In addition, HCK reciprocally phosphorylates TfR‐1 to establish a positive feedback circuit amplifying the prometastatic signalling.

**Conclusions:**

This work identifies a previously uncharacterized prometastatic function of TfR‐1 independent of its canonical iron transport activity in breast cancer. We uncover a reciprocal TfR‑1/HCK positive feedback axis that activates the USP32‑HCK‑STAT3‑MMP9 signalling cascade to facilitate breast cancer metastasis, which provides novel candidate therapeutic targets for metastatic breast cancer intervention.

## Highlights

- TfR‐1 promotes breast cancer lung metastasis independent of its canonical iron transport function.
- TfR‐1 binds HCK via its N‐terminal domain and induces its phosphorylates at Y411.
- TfR‐1 enhances USP32 deubiquitinase activity to stabilize HCK protein.
- TfR‐1‐HCK‐STAT3‐MMP9 axis drives breast tumor growth and metastatic progression.

## Graphical abstract

Schematic summary of TfR‐1‐induced HCK axis activation promoting breast cancer metastasis: TfR‐1 interacts with USP32 and stabilises HCK, directly mediating its phosphorylation and activation. Activated HCK subsequently induces STAT3 phosphorylation and nuclear accumulation, which drives transcriptional upregulation of MMP9, a key mediator of extracellular matrix remodelling and cell migration, ultimately resulting in lung metastasis of breast cancer.

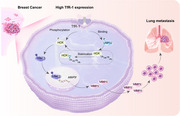

## 1 INTRODUCTION

Breast cancer ranks as the most prevalent malignant tumour type among women in the world.^1^ Advances in early diagnosis, surgical techniques, and hormone and/or targeted therapy have significantly improved the survival rates of breast cancer patients.^2^ Nevertheless, metastatic breast cancer remains an unmet clinical need and is the primary cause of death among breast cancer patients, with a median overall survival of only 2–3 years.^3^ Consequently, elucidating the molecular mechanism underlying breast cancer metastasis is very critical for developing novel therapeutic strategies.

Dysregulation of iron homeostasis plays a critical role in both the initiation and metastasis of breast cancer, and related iron homeostasis regulators are considered potential therapeutic targets for breast cancer, although significant progress has not yet been achieved perhaps due to unclear detailed mechanisms. Our previous study revealed that the lysine methyltransferase G9a suppresses the ferroxidase HEPH, thereby elevating intracellular Fe^2+^ levels and accelerating breast cancer growth.^4^ In this study, we focused on transferrin receptor 1 (TfR‐1), also known as CD71, encoded by the *TFRC* gene, which is the primary mediator of cellular iron uptake and essential for maintaining iron homeostasis.^5^, ^6^ TfR‐1 is a type II transmembrane glycoprotein that forms a homodimer and is widely expressed on the surface of most cells.^6^ Mounting evidence indicates that malignant cells frequently overexpress TfR‐1 and this elevated expression is often associated with poor prognosis across various cancer types.^7^, ^8^ In hepatocellular carcinoma, the upregulated TfR‐1 facilitates cell proliferation and invasion in an iron‐dependent manner by activating the mTOR signalling pathway.^9^ In ovarian cancer tumour tissues, TfR‐1 is highly expressed, inducing ‘iron addiction’ in tumour‐initiating cells that subsequently promotes cells proliferation and peritoneal metastasis.^10^ Studies have also shown that elevated TfR‐1 in breast cancer cell lines (e.g., MCF‐7 and 4T‐1), compared to MCF‐12A human epithelial cells, increases intracellular total iron and promotes lung metastasis,^11^ although the associated regulatory mechanism has not been expounded. Given its high expression on tumour cell surfaces and its distinctive endocytic recycling pathway, TfR‐1 is frequently exploited as a vehicle for anti‐cancer drug‐targeted delivery. However, the development of antibodies or small‐molecule inhibitors that directly target TfR‐1 for anti‐tumour therapy remains a largely underexplored and promising research frontier.

Notably, recent studies have uncovered novel, iron‐transport independent functions of TfR‐1. Evidence indicates that TfR‐1 undergoes nuclear translocation, where it binds to p53 and modulates the expression of genes essential for DNA damage repair.^12^ In addition, Src has been reported to constitutively interact with TfR‐1, and to phosphorylate tyrosine 20 (Tyr20) located within the cytoplasmic domain of TfR‐1. This phosphorylation event confers anti‐apoptotic effects and enhances breast cancer cell survival,^13^ implicating TfR‐1 as a signalling molecule beyond its canonical iron uptake role. While these results suggest a plausible interaction between TfR‐1 and Src‐family kinases (SFKs), whether TfR‐1 serves as a multifunctional regulator in orchestrating oncogenic signalling and breast cancer progression necessitates further mechanistic exploration.

In this study, by integrating human breast cancer patient specimens with complementary in vitro and in vivo preclinical models, we illustrate that elevated TfR‐1 expression drives metastatic dissemination. Mechanistically, we uncover an iron‐independent function of TfR‐1, which facilitates phosphorylation of the SFK member haematopoietic cell kinase (HCK), ultimately enhancing breast cancer metastatic potential. These findings point to TfR‐1 as a potential target for breast cancer therapy and highlight its downstream axis for intervention.

## 2 RESULTS

### 2.1 TfR‐1 overexpression accelerates breast tumour growth and metastasis in preclinical models

**FIGURE 1:**
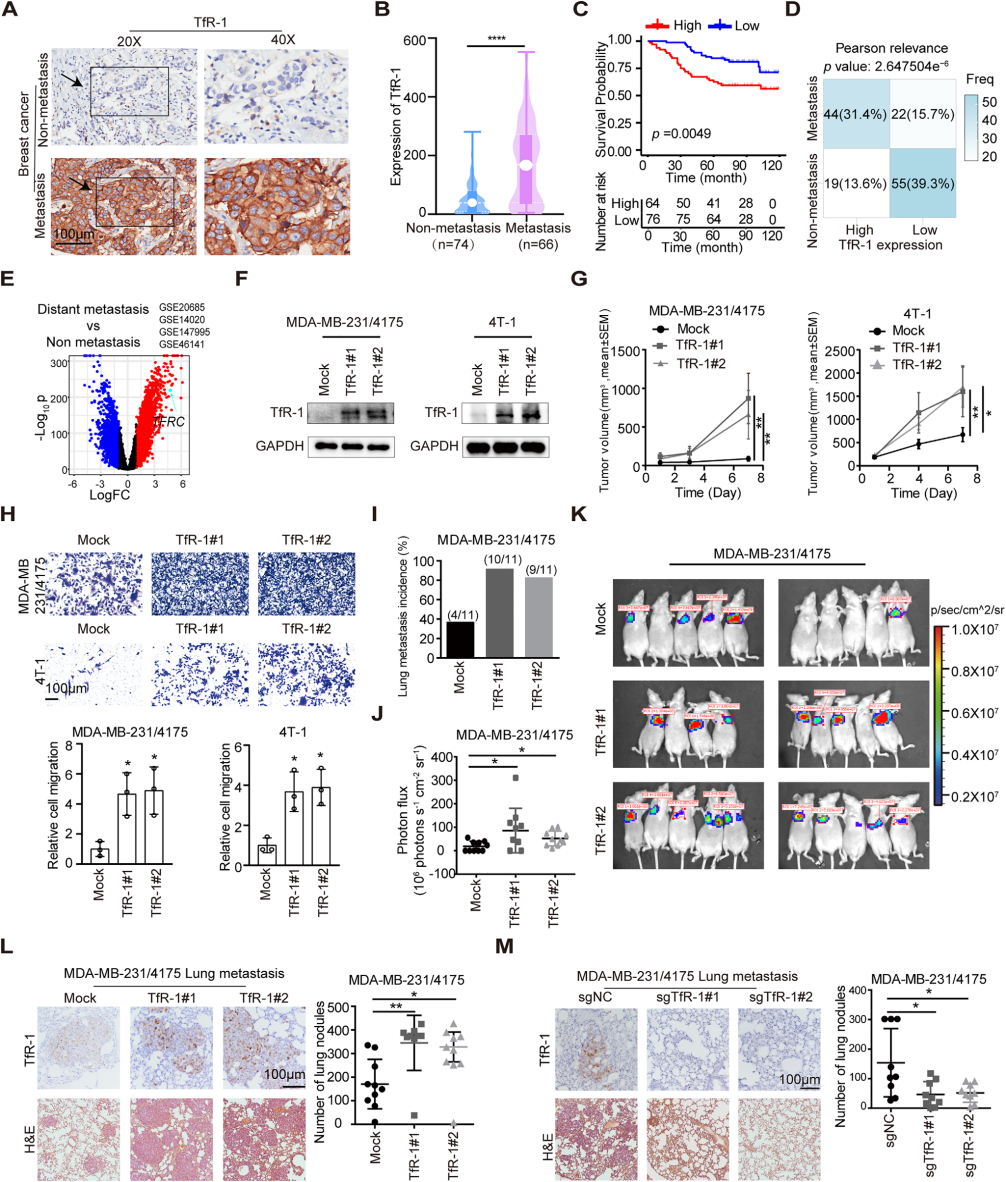
Transferrin receptor 1 (TfR‐1) overexpression accelerates breast tumour growth and metastasis in preclinical models. (A) Immunohistochemical staining of TfR‐1 was performed on tissue arrays consisting of various stages of breast cancer samples. Scale bar: 100 µm. (B) A total of 140 samples was analysed, and the expression of TfR‐1 was determined by evaluating the degree and intensity of immunopositivity. The differences between metastatic and non‐metastatic cancer were assessed using a *t*‐test (^***^ *p* < .0001). (C) Kaplan‒Meier curves of the breast cancer cohort over the expression of TfR‐1. Number at risk tables are shown. (D) The relationship between high expression of TfR‐1 and metastasis, calculated using Pearson correlation analysis. (E) Differential gene expression between primary and metastatic breast cancer evaluated using datasets from the Gene Expression Omnibus (GEO) database (GSE20685, GSE14020, GSE147995 and GSE46141). (F) Western blot (WB) analysis showed TfR‐1 overexpression in MDA‐MB‐231/4175 and 4T‐1 cell lines. (G) Mock or TfR‐1‐overexpressing MDA‐MB‐231/4175 (*n* = 6) and 4T‐1 (*n* = 6) cells were subcutaneously injected. Tumour volume was measured over time. (H) The effect of TfR‐1 on migration in MDA‐MB‐231/4175 and 4T‐1 cells was tested by Transwell assay. Data are expressed as mean ± standard deviation (SD) and representative of at least three independent experiments. (I) The incidence of lung metastasis was assessed 10 days after injection of MDA‐MB‐231/4175 mock and TfR‐1‐overexpressing cells (*n* = 11/group). (J and K) MDA‐MB‐231/4175 mock and TfR‐1‐overexpressing cells were injected into nude mice via tail vein. Bioluminescence images and intensities (p/s/cm^2^/sr) of mice were captured on 19th day after injection (*n* = 10 in mock and TfR‐1#2 group, and *n* = 9 in TfR‐1#1 group). (L and M) Representative haematoxylin and eosin (H&E) and TfR‐1‐immunostained lung metastasis tissue sections are shown, and the number of lung metastasis nodules was counted. Scale bar: 100 µm. Data are presented as mean ± SEM. Statistical significance was assessed using an unpaired, two‐tailed Student's *t*‐test, ^*^ *p* < .05, ^**^ *p* < .01, ^***^ *p* < .001. The statistical significance was calculated compared to mock in each group.

To assess the clinical relevance of TfR‐1 in breast cancer, immunohistochemical staining was performed on a tissue microarray comprising 66 metastatic and 74 non‐metastatic human breast tumour samples. The results demonstrated that the level of TfR‐1 was significantly higher in metastatic tumour tissues than that in the non‐metastatic cohort (Figure 1A,B). The LightGBM model yielded the best area under the curve (AUC) value of .94543 for TfR‐1 expression in these 140 patient tumour samples, with an optimal cutoff value of 75 (*H*‐score) (Figure S1A). Compared with the low TfR‐1 group, the survival period of the high TfR‐1 expression group was significantly shortened (Figure 1C). And more importantly, we also observed a strong positive correlation between a high level of TfR‐1 and metastasis incidence (Figure 1D). We conducted further investigations using the public Gene Expression Omnibus (GEO) database, and revealed that the level of TfR‐1 was dramatically upregulated in metastatic breast cancer tissues compared to that in primary tissues (Figure 1E). Altogether, these data strongly suggest that the high expression of TfR‐1 is positively correlated with the poor prognosis of human breast cancer and the occurrence of metastasis. Therefore, elucidating the precise role of TfR‐1 in breast cancer progression is imperative.

Using a lentiviral vector, we stably overexpressed TfR‐1 in three human breast cancer cell lines: MCF‐7, MDA‐MB‐231, and a well‐characterised luciferase tagged lung‐selective metastatic MDA‐MB‐231 variant MDA‐MB‐231/4175^14^ and one mouse breast cancer cell line 4T‐1 (Figures 1F and S1B). We found that TfR‐1 overexpression markedly enhanced breast cancer cells proliferation in vitro (Figure S1C) and promoted tumour growth in vivo (Figure 1G). Furthermore, Transwell assay results confirmed our clinical findings, demonstrating that overexpressed TfR‐1 significantly enhanced the migration capacity of these cell lines compared to control ones (Figures 1H and S1D). To assess metastatic potential in vivo, we intravenously injected TfR‐1‐overexpressing MDA‐MB‐231/4175 cells into nude mice. Compared with the mock group, these mice exhibited remarkably enhanced lung metastatic incidence (Figure 1I). Then, bioluminescence imaging monitoring was further used and revealed increased metastatic outgrowth in the lungs of mice intravenously injected with TfR‐1‐overexpressing MDA‐MB‐231/4175 cells (Figure 1J,K). And histological analyses confirmed that a high level of TfR‐1 dramatically enhanced the average number and area of lung metastatic nodules (Figure 1L). Importantly, the spontaneous metastasis model using TfR‐1‐overexpressing mouse breast cancer cell line 4T‐1 inoculated into mammary fat pads of mice showed a significantly larger average number of lung micrometastatic nodules at approximately 3 weeks compared to controls (Figure S1E).

Furthermore, we generated TfR‐1‐knockdown MDA‐MB‐231/4175 and MDA‐MB‐231 cells using the CRISPR/Cas9 system (Figure S1F). TfR‐1 depletion dramatically impaired cell migration (Figure S1G). Likewise, intravenous injection of TfR‐1‐depleted MDA‐MB‐231/4175 cells into nude mice resulted in significantly fewer metastatic lung tumour colonies (Figure 1M). Collectively, these data establish a critical role for TfR‐1 in breast cancer progression, particularly in promoting breast cancer cells metastasis.

### 2.2 TfR‐1 activates the HCK‒STAT3 signalling axis

**FIGURE 2:**
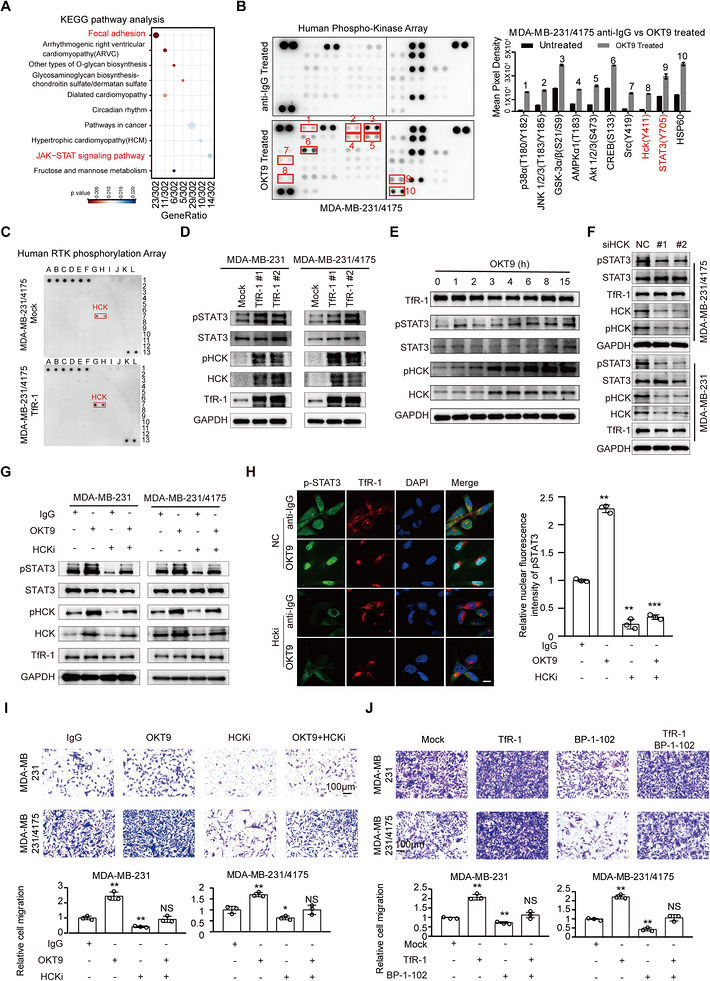
Transferrin receptor 1 (TfR‐1) activates the haematopoietic cell kinase (HCK)‒STAT3 signalling axis. (A) Kyoto Encyclopedia of Genes and Genomes (KEGG) enrichment analysis of the differentially expressed genes between mock and TfR‐1‐overexpressing MDA‐MB‐231/4175 cell lines. (B) MDA‐MB‐231/4175 cells were treated with OKT9 (1 µg/mL), and subjected to human phospho‐kinase array assay (R&D system). The corresponding upregulated kinases were shown in red frames. (C) MDA‐MB‐231/4175 mock and TfR‐1‐overexpressing cells were analysed using a human RTK phosphorylation antibody array C1 (Ray Biotech). The corresponding upregulated kinases were shown in red frames. (D) The expression of specific protein was assessed by Western blot (WB) in lysates from mock or TfR‐1‐overexpressing cells. (E) WB analysis of MDA‐MB‐231/4175 cells treated with OKT9 at indicated time points. (F) WB analysis of lysates from MDA‐MB‐231 and MDA‐MB‐231/4175 cells transfected with siNC or HCK‐targeting small interfering RNA (siRNA). (G) Immunoblotting of MDA‐MB‐231 and MDA‐MB‐231/4175 cells pretreated with or without HCK inhibitor RK‐20449 (.1 µM, 24 h) followed by OKT9. (H) Phosphorylation of STAT3 in cells was subsequently examined by immunofluorescence microscopy in MDA‐MB‐231/4175 cells treated as indicated. Scale bar represents 10 µm. (I) Transwell assay of indicated treatment in MDA‐MB‐231 and MDA‐MB‐231/4175 cells. (J) Migration of MDA‐MB‐231 and MDA‐MB‐231/4175 mock or TfR‐1‐overexpressing cells after 24‐h treatment with BP‐1‐102 (10 µM, STAT3 inhibitor) were assessed by Transwell assay. Data are shown as mean ± standard deviation (SD) from at least three independent experiments. Statistical significance was determined by unpaired, two‐tailed Student's *t*‐test (^*^ *p* < .05, ^**^ *p* < .01, ^***^ *p* < .001) versus respective controls.

Furthermore, RNA sequencing was performed on mock and TfR‐1‐overexpressing MDA‐MB‐231/4175 cell lines to elucidate the mechanism underlying TfR‐1's pro‐metastatic role. Kyoto Encyclopedia of Genes and Genomes (KEGG) pathway enrichment analysis of differentially expressed genes revealed a significant enrichment of focal adhesion and the JAK‒STAT signalling pathway, which has been recognised as a key regulator of tumour metastasis^15^ (Figure 2A). Consistent with this finding, Western blot (WB) analysis showed that overexpressed TfR‐1 significantly increased the phosphorylation of STAT3 without altering total STAT3 protein levels, and accelerated its nuclear translocation (Figure S2A). Treatment with OKT9, a monoclonal antibody targeting the apical domain of human TfR‐1^16^ also induced STAT3 phosphorylation (Figure S2B) and promoted breast cancer cells migration (Figure S2C), mimicking the effects of TfR‐1 overexpression. To identify upstream signals responsible for STAT3 phosphorylation, a phospho‐kinase antibody array was performed following OKT9 treatment. The assay revealed significant increases in phosphorylation of multiple kinases, including HCK (Y411), STAT3 (Y705), Src (Y419), p38α (T180/Y182), JNK 1/2/3 (T183/Y185), GSK‐3α/β (S21/S9), AMPKα1 (T183), Akt 1/2/3 (S473), CREB (S133) and HSP60 (Figure 2B). Meanwhile, among the 71 RTKs screened using the Human RTK Phosphorylation Antibody Array C1, only HCK, a member of SFK, showed elevated phosphorylation in TfR‐1‐overexpressing MDA‐MB‐231/4175 cells (Figure 2C). And RNA‐seq heatmap analysis revealed transcriptional upregulation of SHC2 (Sck), a well‐known adaptor molecule containing Src homology 2 (SH2) domain^17^ in TfR‐1‐overexpressing cells (Figure S2D), further supporting potential cooperation between TfR‐1 and tyrosine kinases. Then, an increase in HCK phosphorylation was confirmed in TfR‐1‐overexpressing MDA‐MB‐231/4175 cells by WB (Figure 2D). Notably, the total level of HCK was also unexpectedly increased concomitantly (Figure 2D). Meanwhile, OKT9 also induced remarkably elevated HCK phosphorylation in MDA‐MB‐231/4175 cells, along with an increase in basal HCK (Figure 2E).

Furthermore, both small interfering RNA (siRNA)‐mediated HCK knockdown and pharmacological inhibition using HCK inhibitor (HCKi) RK‐20449 remarkably attenuated the increased phosphorylation of HCK and STAT3, and reversed the effect of OKT9, underscoring HCK as a pivotal mediator (Figures 2F,G and S2E). Cellular immunofluorescence analysis confirmed that HCK inhibition suppressed OKT9‐induced STAT3 phosphorylation and translocation into the nucleus (Figure 2H). Both HCKi and STAT3 inhibitor BP‐1‐102 effectively impeded breast cancer cell migration and reversed the pro‐metastatic effects of either OKT9 or TfR‐1 overexpression (Figure 2I,J). While BP‐1‐102 significantly reduced OKT9‐induced STAT3 phosphorylation and cell migration, it did not affect TfR‐1 and HCK phosphorylation, suggesting that STAT3 acts as a downstream effector of HCK signalling (Figure S2F,G). These results identified the HCK‒STAT3 axis as a significant driver of TfR‐1‐mediated breast cancer metastasis.

### 2.3 TfR‐1 promotes HCK phosphorylation via its N‐terminus

**FIGURE 3:**
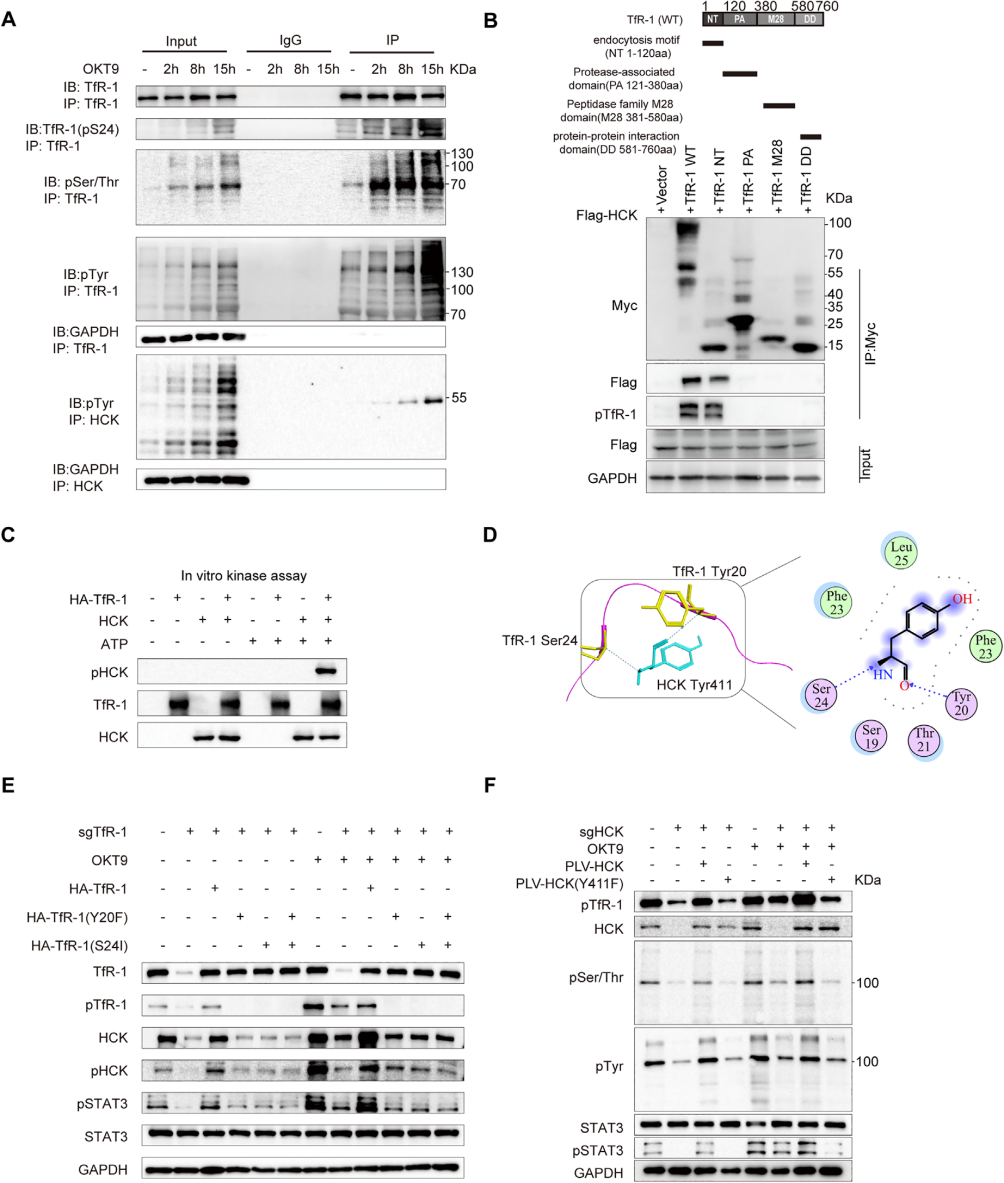
Transferrin receptor 1 (TfR‐1) promotes haematopoietic cell kinase (HCK) phosphorylation via its N‐terminus. (A) Anti‐TfR‐1 immunoprecipitates from MDA‐MB‐231/4175 cells with or without OKT9 stimulation for indicated times were analysed by Western blot (WB) using pan‐phospho‐Ser/Thr antibody and anti‐HCK immunoprecipitates were analysed using pan‐phospho‐tyrosine antibody. (B) HEK293T cells transfected with plasmids encoding Flag‐HCK and Myc‐tagged TfR‐1 wild‐type (WT) or truncation mutants for 24 h were subjected to immunoprecipitation (anti‐Myc) and immunoblot analysis (anti‐Myc or anti‐Flag). (C) In vitro kinase assay assessed HCK phosphorylation. HA‐TfR‐1 immunoprecipitated from HEK293T cells transfected with pcDNA3.2/DEST/hTfR‐HA was incubated with purified HCK with or without ATP. pHCK was detected by WB. (D) Molecular docking assessed binding affinity between HCK (AlphaFoldDB: P08631) and TfR‐1 (AlphaFoldDB: P02786), yielding a binding energy of ‒3.3816 kcal/mol, indicating moderate stability. (E) WB analysis of total lysates from MDA‐MB‐231/4175 sgTfR‐1 cells transfected with HA‐TfR‐1 WT or Y20F/S24I variant plasmids and treated with or without OKT9. (F) The effect of OKT9 on the signalling pathway in MDA‐MB‐231/4175 cells with sgHCK after transfection with either HCK WT or Y411F mutant plasmids.

Subsequently, we performed a series of co‐immunoprecipitation (Co‐IP) assays to elucidate how TfR‐1 activates HCK. Our initial results demonstrated that OKT9 promoted the phosphorylation of TfR‐1 while simultaneously enhancing the tyrosine phosphorylation of HCK in MDA‐MB‐231/4175 cells (Figure 3A). And phosphorylated TfR‐1 co‐immunoprecipitated with upregulated HCK and its phosphorylated form at residue Y411 (Figure S3A). Given prior reports that TfR‐1‐Src complex formation induces the phosphorylation of TfR‐1 at Tyr20 (Y20),^13^ we next attempted to identify the domain of TfR‐1 mediating the interaction with HCK. We co‐expressed Flag‐tagged HCK with truncated Myc‐tagged TfR‐1 constructs (TfR‐1‐NT: N‐terminus, TfR‐1‐PA: protease‐associated domain, TfR‐1‐M28: peptidase family M28 domain and TfR‐1‐DD: dimerisation domain) in HEK293T cells. Co‐IP revealed that the N‐terminus domain of TfR‐1 is essential for its physical interaction with HCK and HCK phosphorylation (Figure 3B). Critically, in vitro kinase assays demonstrated that HA‐TfR‐1 increases phosphorylation of purified HCK at Y411 (Figure 3C). Molecular docking further indicated potential physical interactions between TfR‐1 (Y20/S24) and HCK Y411 (Figure 3D), supporting the mechanism. Moreover, CRISPR/Cas9‐mediated HCK deletion blunted the global OKT9‐induced tyrosine phosphorylation of TfR‐1 (Figure S3B,C), revealing a feedback loop.

Previous studies show that growth factors such as phorbol 12‐myristate 13‐acetate or platelet‐derived growth factor could cause a marked increase in TfR‐1 S24 phosphorylation, which is also the major site phosphorylated by protein kinase C.^18^, ^19^ In order to test functional reciprocity, we reconstituted TfR‐1‐knockdown cells with phosphorylation‐defective mutants. Cells expressing TfR‐1 Y20F or S24I variants, alone or in combination, exhibited markedly attenuated OKT9‐induced HCK tyrosine phosphorylation, compared to cells expressing wild‐type (WT) TfR‐1 (Figure 3E). Similarly, cells expressing HCK Y411F mutant displayed diminished phosphorylation of TfR‐1 and mitigated response to OKT9 (Figure 3F). Furthermore, both TfR‐1 phospho‐mutations (Y20F and/or S24I) and HCK Y411F mutation impaired STAT3 phosphorylation, indicating functional disruptions of this axis (Figure 3E,F). Collectively, these data demonstrate that TfR‐1 promotes HCK phosphorylation at Y411 via its N‐terminus, and a potential reciprocal positive feedback loop between HCK and TfR‐1, ultimately amplifies STAT3 activation.

### 2.4 TfR‐1 stabilises HCK protein through deubiquitinase ubiquitin‐specific protease 32

**FIGURE 4:**
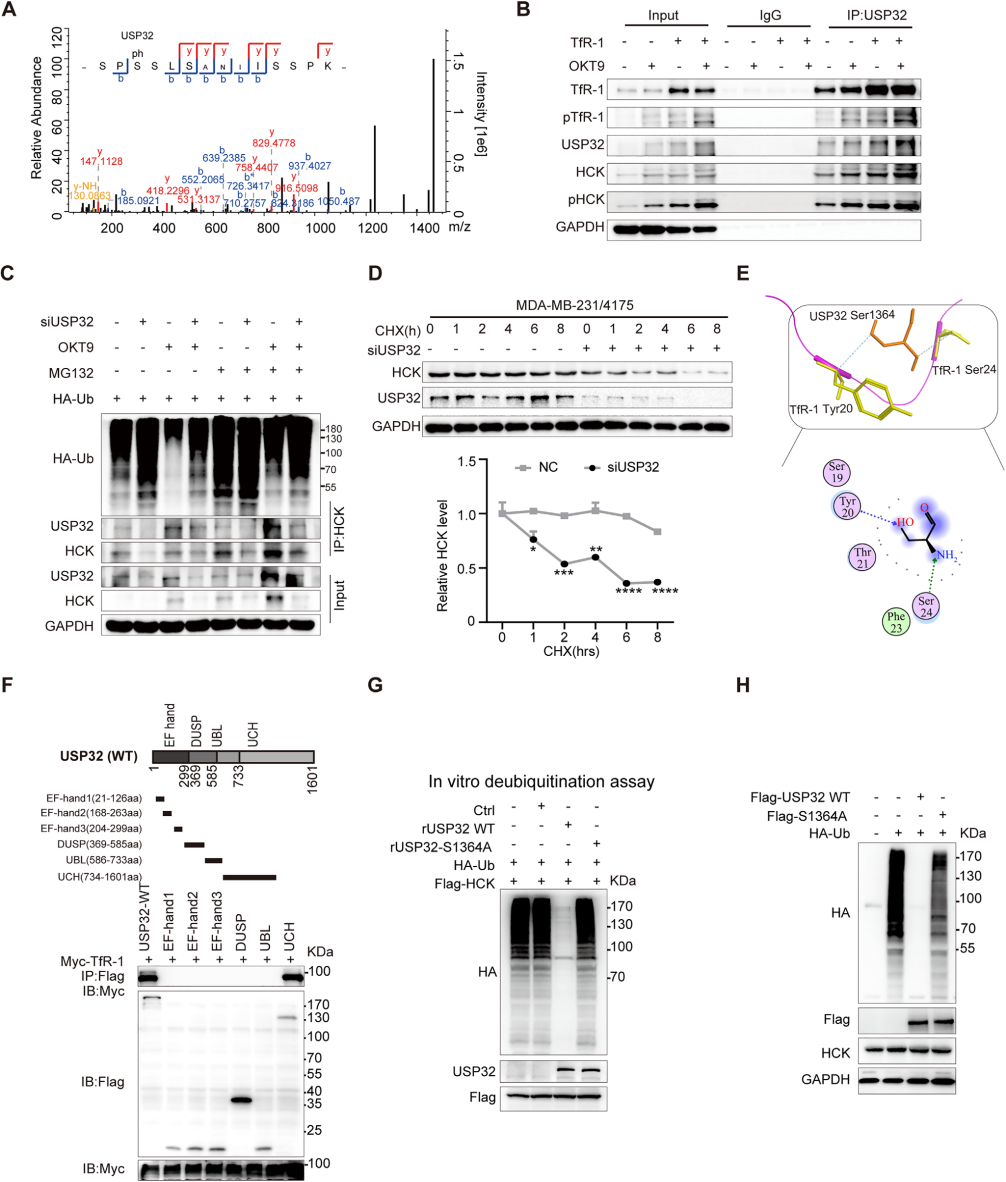
Transferrin receptor 1 (TfR‐1) stabilises haematopoietic cell kinase (HCK) protein through deubiquitinase USP32. (A) Mass spectrometry analysis of endogenous TfR‐1 immunopurified from MDA‐MB‐231/4175 cells revealed phosphorylation of USP32 at S1364. (B) Endogenous co‐immunoprecipitation (Co‐IP) in MDA‐MB‐231/4175 cells with anti‐USP32 antibody, followed by Western blot (WB) against TfR‐1, pTfR‐1, HCK and pHCK. (C) MDA‐MB‐231/4175 cells co‐transfected with HA‐ubiquitin plasmid and USP32 small interfering RNAs (siRNAs) were treated with OKT9 or 10 µM MG132 for 6 h before collection. Ubiquitinated HCK level was detected after immunoprecipitation with HCK. (D) MDA‐MB‐231/4175 cells transfected with siNC or siUSP32 were treated with 100 µg/mL CHX for indicated time to detect HCK stability. HCK intensities normalised to GAPDH were quantified by ImageJ. (E) Molecular docking analysis revealed a direct interaction between TfR‐1 (AlphaFoldDB: P02786) and USP32 (AlphaFoldDB: Q8NFA0), with a calculated binding energy of –3.1043 kcal/mol. (F) HEK293T cells were co‐transfected with Myc‐TfR‐1 and different Flag‐tagged USP32 domains (EF‐hand1 [21‒126 aa], EF‐hand2 [168‒263 aa], EF‐hand3 [204‒299 aa], DUSP [369‒585 aa], UBL [586‒733 aa] and UCH [734‒1601 aa]) to identify the interacting domains. (G) In vitro assay disclosed that bacterial‐expressed recombinant human USP32, but not its S1364A mutant, effectively deubiquitinated HCK. (H) Total ubiquitination level of HCK was detected by WB in HEK293T cells transfected with wild‐type (WT) or S1364A‐mutated USP32. Data are shown as mean ± standard deviation (SD) from at least three independent experiments. Statistical significance was determined by unpaired, two‐tailed Student's *t*‐test (^*^ *p* < .05, ^**^ *p* < .01, ^***^ *p* < .001, ^****^ *p* < .0001) versus respective controls.

Thus far, it was still confusing to us why TfR‐1 stimulation dramatically elevated the basal level of HCK in such a short time. We noticed a rapid increase in HCK protein levels following TfR‐1 phosphorylation, without a corresponding change in HCK mRNA (Figure S4A), suggesting a post‐transcriptional regulation of HCK stability. To explore the mechanism, affinity purification coupled with liquid chromatography/tandem mass spectrometry (LC‒MS/MS) was employed to analyse endogenous TfR‐1‐interacting proteins in OKT9‐stimulated MDA‐MB‐231/4175 cells. Although previous studies have demonstrated that HCK is subject to ubiquitination, the specific deubiquitinating enzyme responsible for this process has not yet been identified.^20^ Here, we identified a peptide indicating the phosphorylation of ubiquitin‐specific protease 32 (USP32) at Ser1364 (Figure 4A). Then, the presence of the USP32 in the TfR‐1 interactome was confirmed by WB using the co‐immunoprecipitated proteins, which revealed increased endogenous binding among USP32, TfR‐1 and HCK in OKT9‐treated cells compared to controls (Figure 4B). Since USP32 functions as a deubiquitinase,^21^ we further investigated its impact on HCK ubiquitination. In MDA‐MB‐231/4175 cells co‐expressing HA‐ubiquitin and exposed to MG132, TfR‐1 phosphorylation markedly dampened HCK ubiquitination, whereas USP32 knockdown reversed this effect and boosted HCK ubiquitination levels (Figure 4C). Furthermore, cycloheximide (CHX) chase assays demonstrated that USP32 depletion shortened the half‐life of endogenous HCK (Figure 4D).

Molecular docking analyses further suggested that the interaction between USP32 and TfR‐1 was possibly mediated via the Ser1364 residue of USP32 and the Tyr20/Ser24 residues of TfR‐1 (Figure 4E). To pinpoint the region of USP32 that engages TfR‐1, we constructed six N‐terminal Flag‐tagged truncation fragments: EF‐hand1 (21‒126 aa), EF‐hand2 (168‒263 aa), EF‐hand3 (204‒299 aa), DUSP (369‒585 aa), UBL (586‒733 aa) and UCH (734‒1601aa). Co‐IP mapping revealed that only full‐length USP32 and its UCH domain specifically interact with TfR‐1 (Figure 4F). Co‐IP analyses with Myc‐tagged WT and truncated TfR‐1 constructs further showed that USP32 co‐precipitated exclusively with full‐length TfR‐1 and its N‐terminal region, a finding consistent with the TfR‐1‐binding profile previously observed for HCK (Figure S4B).

Next, site‐directed mutagenesis was used to generate the USP32 S1364A mutant to validate the requirement of phosphorylation of USP32 at Ser1364 for its deubiquitinase activity towards HCK. This mutation was predicted to disrupt the catalytic core based on a structural comparison of the putative USP domains of DUBs. Subsequent in vitro deubiquitination assays using purified recombinant proteins confirmed that WT USP32, but not the phospho‐deficient mutant (S1364A), markedly deubiquitinated HCK (Figure 4G). To evaluate this activity in cells, we transfected MDA‐MB‐231/4175 cells with HA‐ubiquitin together with either USP32 WT or the S1364A mutant plasmids, immunoprecipitated HCK, and detected its ubiquitylation. The results revealed that the USP32 S1364A mutant failed to deubiquitylate HCK (Figure 4H). Furthermore, by investigating the role of USP32 in breast cancer cells mobility in vitro, we found that USP32 knockdown dramatically suppressed the activation of HCK‒STAT3 signalling, reduced the migration potential of MDA‐MB‐231/4175 cells, and counteracted the effects induced by OKT9 (Figure S4C,D). Together, these findings suggest that TfR‐1 interacts with USP32 and facilitates USP32‐mediated HCK deubiquitination, which may contribute to HCK stabilisation and activation of downstream signalling pathways.

### 2.5 Matrix metalloproteinase 9 is a central downstream executor in TfR‐1‐promoted breast cancer cells mobility

**FIGURE 5:**
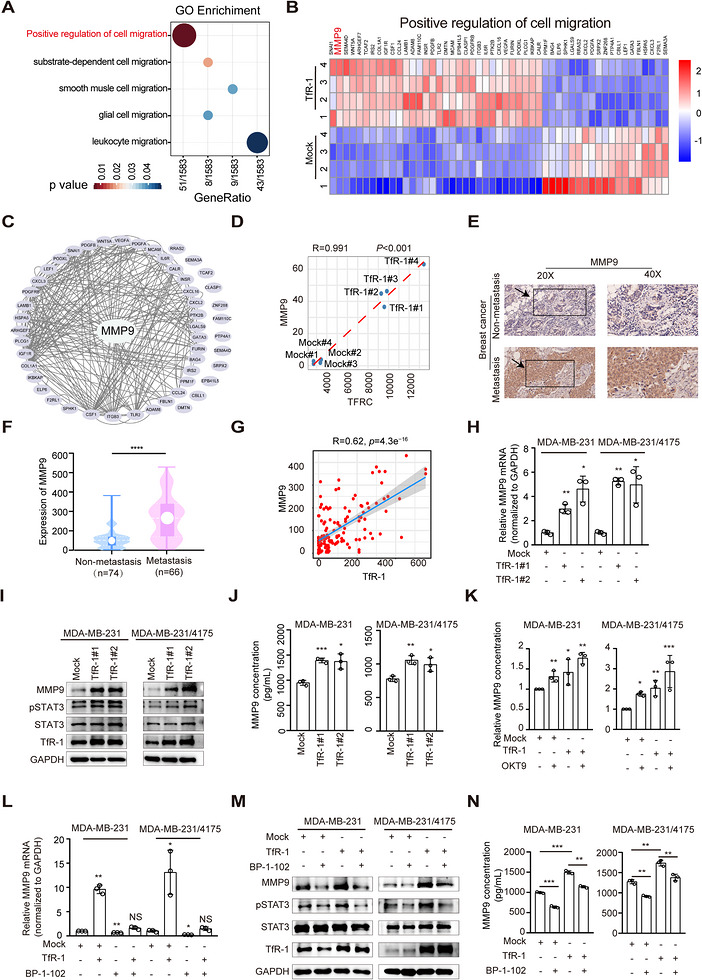
Matrix metalloproteinase 9 (MMP9) is a central downstream executor in transferrin receptor 1 (TfR‐1)‐promoted breast cancer cells mobility. (A) Differentially expressed genes between mock and TfR‐1‐overexpressed MDA‐MB‐231/4175 cells were analysed by Gene Ontology (GO) enrichment. (B) Heatmap depicting gene expression in positive regulation of cell migration pathways for mock versus TfR‐1‐overexpressing MDA‐MB‐231/4175 cells. (C) Core genes in migration‐related pathways were evaluated using degree, betweenness and closeness algorithms. (D) Pearson correlation analysis revealed strong positive correlation between *TFRC* and *MMP9* expression (*R* = .991, *p* < .001). (E) Expression of MMP9 was detected by immunohistochemical (IHC) in 74 non‐metastatic and 66 metastatic breast cancer tissue samples. (F) The expression of MMP9 in the breast cancer tissue microarray was quantified based on staining intensity and extent. (G) Correlation analysis was performed to evaluate the relationship between the expression levels of TfR‐1 and MMP9 in breast cancer tissues. (H) Relative mRNA expression levels of *MMP9* in MDA‐MB‐231 and MDA‐MB‐231/4175 mock or TfR‐1‐overexpressing cells were determined by real‐time quantitative PCR (RT‐qPCR). (I) Western blot (WB) analysis of total STAT3, pSTAT3 and MMP9 in MDA‐MB‐231 and MDA‐MB‐231/4175 mock or TfR‐1‐overexpressing cells. (J) MMP9 secretion in culture supernatants was measured by enzyme‐linked immunosorbent assay (ELISA) in MDA‐MB‐231 and MDA‐MB‐231/4175 mock or TfR‐1‐overexpressing cells. (K) ELISA analysis of MMP9 secretion in supernatants from TfR‐1‐overexpressing MDA‐MB‐231 and MDA‐MB‐231/4175 cells treated with or without OKT9. (L) RT‐qPCR analysis of *MMP9* mRNA in TfR‐1‐overexpressing cells treated with or without STAT3 inhibitor BP‐1‐102. (M) WB analysis of MMP9, pSTAT3, STAT3 and TfR‐1 in MDA‐MB‐231 and MDA‐MB‐231/4175 TfR‐1‐overexpressing cells with or without BP‐1‐102. (N) ELISA measurement of MMP9 secretion in TfR‐1‐overexpressing MDA‐MB‐231 and MDA‐MB‐231/4175 cells treated with or without BP‐1‐102. Data represent mean ± standard deviation (SD) from at least three independent experiments. Statistical significance was determined by unpaired, two‐tailed Student's *t*‐test, ^*^ *p* < .05, ^**^ *p* < .01, ^***^ *p* < .001, with comparisons made to relevant controls in each experiment.

We then conducted analysis using RNA‐seq data from TfR‐1‐overexpressing and mock MDA‐MB‐231/4175 cells to determine how TfR‐1/HCK/STAT3 accelerated tumour metastasis. Gene Ontology (GO) enrichment analysis revealed significant enrichment of cell migration‐related pathways (Figure 5A). These migration‐associated genes, *MMP9*, *VEGFA*, *PDGFRB* and *SEMA4D*, were significantly upregulated in TfR‐1‐overexpressing cells (Figure 5B). Employing network centrality measures (degree, betweenness and closeness analyses), we pinpointed matrix metalloproteinase 9 (MMP9) as the central effector (Figure 5C). Notably, a strong positive correlation was observed between the level of *TFRC* and *MMP9* in these cells (Figure 5D). In addition, a prior research has identified elevated serum MMP9 as a biomarker for distant dissemination in breast cancer patients with brain metastases.^22^ Consistently, in our breast cancer patient cohort, immunohistochemical (IHC) analysis revealed that MMP9 expression was also upregulated in metastatic samples, and was positively correlated with TfR‐1 expression (Figure 5E‒G). All these supports our further confirmation of the crucial role of MMP9 in TfR‐1‐induced breast cancer metastasis. As expected, overexpression of TfR‐1 in MDA‐MB‐231 and MDA‐MB‐231/4175 cell lines distinctly increased both intracellular MMP9 expression and its extracellular secretion (Figure 5H‒J). Conversely, knockdown of TfR‐1 simultaneously decreased intracellular and extracellular MMP9 (Figure S5A,B). Moreover, treatment with OKT9 also significantly enhanced MMP9 expression and secretion (Figures 5K and S5C). Consistent with the previous report,^23^ STAT3 inhibitor BP‐1‐102 effectively suppressed MMP9 transcriptional activation and protein expression (Figure 5L‒N). USP32 knockdown also significantly reversed the OKT9‐mediated upregulation of MMP9 in these breast cancer cell lines (Figure S5D). Although TfR‐1 is a well‐established regulator of iron homeostasis^12^ treatment with the iron chelator deferoxamine (DFO) did not impair TfR‐1‐mediated activation of the HCK‒STAT3 signalling pathway (Figure S5E). These findings suggest that TfR‐1‐mediated regulation of the HCK‒STAT3 signalling pathway is independent of iron‐related mechanisms.

**FIGURE 6:**
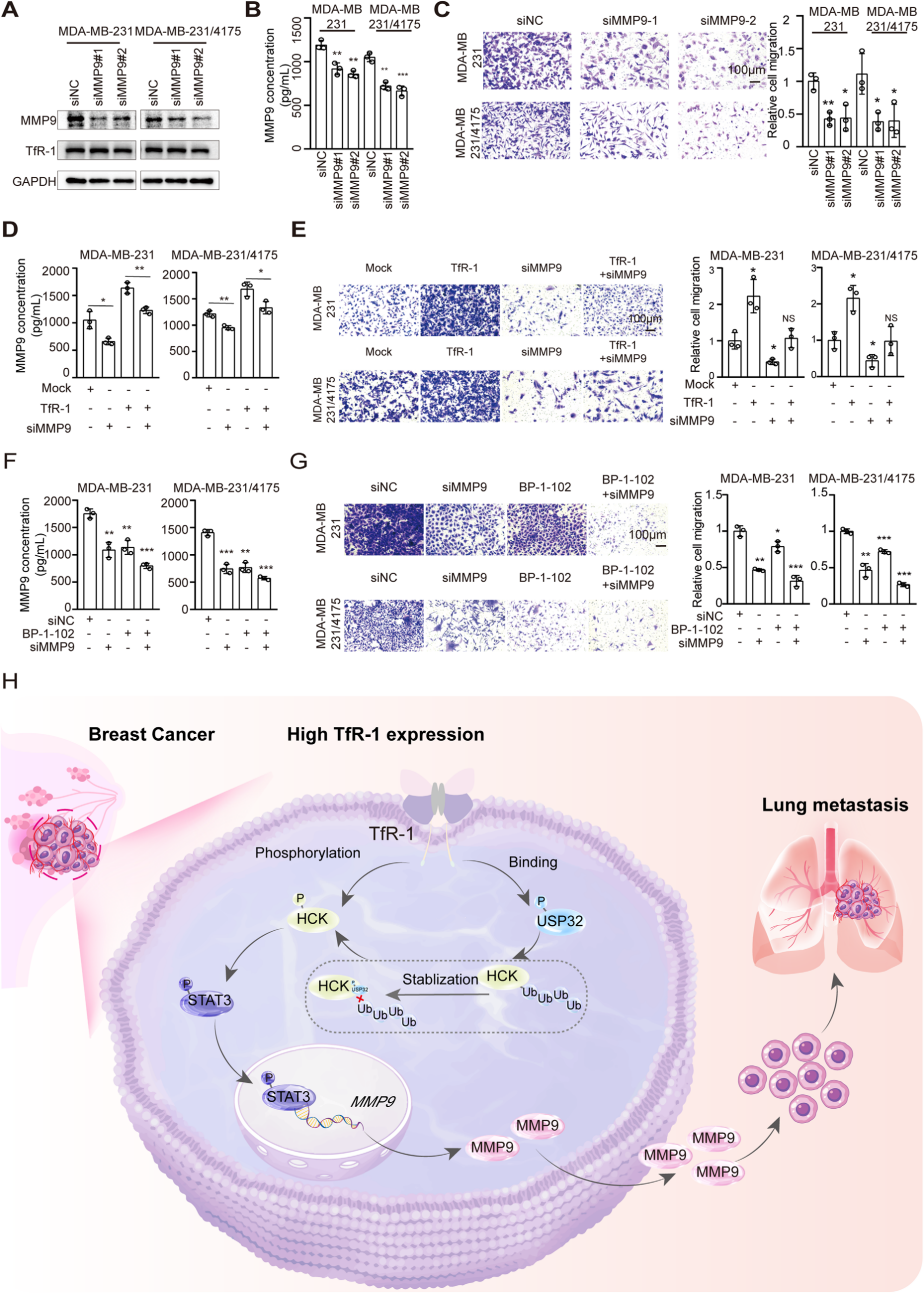
STAT3‒matrix metalloproteinase 9 (MMP9) signalling mediates the pro‐metastatic effects of transferrin receptor 1 (TfR‐1) in breast cancer. (A) Western blot (WB) analysis of TfR‐1 and MMP9 expression in MMP9 depletion breast cancer cells. (B) Enzyme‐linked immunosorbent assay (ELISA) analysis of secreted MMP9 levels after siMMP9 transfection. (C) Transwell assays comparing siNC‐ or siMMP9‐treated cells. (D) ELISA analysis of secreted MMP9 in mock versus TfR‐1‐overexpressing breast cancer cells transfected with siNC or siMMP9. (E) Transwell assays of mock versus TfR‐1‐overexpressing cells transfected with siNC or siMMP9. (F) Effects of MMP9 knockdown and STAT3 inhibition (BP‐1‐102, 10 µM, 24 h) on MMP9 secretion. (G) Transwell assays of siNC‐ and siMMP9‐treated cells with or without STAT3 inhibitor BP‐1‐102 (10 µM, 24 h). Data are shown as mean ± standard deviation (SD) from at least three independent experiments. Statistical significance was determined by unpaired, two‐tailed Student's *t*‐test, ^*^ *p* < .05, ^**^ *p* < .01, ^***^ *p* < .001, with comparisons made to relevant controls in each experiment. (H) Schematic summary of TfR‐1‐induced haematopoietic cell kinase (HCK) axis activation promoting breast cancer metastasis.

Furthermore, compared to controls, siRNAs targeting MMP9 significantly suppress both MMP9 expression and secretion in MDA‐MB‐231 cells and MDA‐MB‐231/4175 cells (Figure 6A,B), correspondingly dramatically reduced cells migration (Figure 6C). More importantly, loss of MMP9 abolished TfR‐1‐overexpressing‐induced enhancement of cellular migration (Figure 6D,E). Compared to either single treatment, the combination of MMP9 knockdown and BP‐1‐102 synergistically reduced secreted MMP9 (Figure 6F) and suppressed breast cancer cells metastatic potential (Figure 6G). Collectively, these findings indicate that MMP9 critically mediates TfR‐1‐HCK‐STAT3 signalling to drive cells migratory processes, establishing this pathway as a highly druggable target for breast cancer metastasis control.

## 3 DISCUSSION

TfR‐1 is believed to mediate cellular iron uptake through transferrin binding. While its overexpression in various cancer types is well‐documented,^24^, ^25^, ^26^, ^27^ the prevailing view holds that TfR‐1 participates in cancer progression primarily through its canonical iron uptake function. Our previous work also demonstrated that TfR‐1‐mediated disruption of iron homeostasis confers resistance to EZH2 inhibitors in diffuse large B‐cell lymphoma, emphasising the value of TfR‐1 in lymphoma progression.^28^ Nevertheless, whether TfR‐1 possesses non‐iron‐dependent functions and how such functions might contribute to tumours progression remain poorly characterised. In the present study, using clinical samples and preclinical models, we revealed for the first time that elevated TfR‐1 promotes breast cancer progression, notably by enhancing cellular motility and metastasis formation. Crucially, this pro‐metastatic effect depends on the activation of HCK‒STAT3‒MMP9, beyond TfR‐1's canonical function. Specifically, TfR‐1 stabilised and promotes HCK phosphorylation, which subsequently increased STAT3 phosphorylation and nuclear accumulation, leading to transcriptional activation of MMP9, and accelerated cellular motility in breast cancer (Figure 6H). These findings provide a precise mechanistic explanation for TfR‐1‐mediated promotion of breast cancer metastasis, indicating important roles of TfR‐1 beyond iron transport. We emphasise the significance of TfR‐1's phosphorylation function and suggest new potential therapeutic strategies for breast cancer.

Due to critical function in iron‐dependent cell proliferation, and its cell‐surface accessibility role in endocytosis, TfR‐1 presents as a promising anti‐tumour target for cancer therapy over the years. And therapeutic TfR‐1 antibodies are believed to induce iron deprivation, which can directly inhibit proliferation, trigger apoptosis and/or sensitise cells to other anti‐cancer agents.^29^ The mouse monoclonal antibody mAb A24 against TfR‐1 was reported to attenuate proliferation and induce apoptosis in chronic and acute adult T‐cell leukaemia/lymphoma.^30^ Antibodies engineered with a monovalent TfR‐1‐binding site, termed antibody transport vehicle, has been proved to facilitate blood‒brain barrier transcytosis.^31^ However, current interests primarily focus on the utility for targeting delivery in antibody‒drug conjugate drugs due to its high abundance on tumour cell membranes, the efficacy of TfR‐1‐targeting antibodies as direct anti‐cancer agents remains unproven in clinical. Our data further support TfR‐1 as an attractive therapeutic target for breast cancer, particularly metastatic disease. We uncover a novel, non‐canonical kinase‐like activity of TfR‐1 in phosphorylating proteins such as HCK and USP32, suggesting a potential therapeutic strategy of designing TfR‐1 inhibitors to impair this phosphorylation activity.

Another important finding in this study is addressed that HCK serves as the critical functional mediator in TfR‐1 promoted metastasis. HCK, a non‐receptor tyrosine kinase and a member of SFKs alongside Blk, Fyn and Src^32^ was initially characterised for its expression in myeloid cells and its role in innate immune signalling within neutrophils and macrophages, with established links to myeloid leukaemias.^33^ An increasing number of studies have shown that HCK is overexpressed in a variety of tumours, including breast, colon and gastric cancer, correlates with poor prognosis.^34^, ^35^, ^36^ It not only directly impacts cancer cells proliferation, migration and apoptosis, but also influences the tumour microenvironment to promote tumour progression. In glioblastoma (GBM), HCK mediates epithelial‒mesenchymal transition through the TGF‐β/Smad signalling pathway, enhancing GBM cells proliferation and invasion.^37^ Additionally, the heparin binding‐epidermal growth factor‐like (HB‐EGF) growth factor promotes cell growth and migration by phosphorylating HCK, subsequently activating AKT (protein kinase B) and extracellular signal‑regulated kinase signalling.^38^ HCK transcription and activation are also induced by mutant MYD88, positioning it as a key determinant of pro‐survival signalling.^39^ In our study, interactomics and phosphorylation arrays identified that TfR‐1 enhances HCK phosphorylation, subsequently increases STAT3 phosphorylation, enhancing the expression of matrix metalloproteinase MMP9 and accelerating tumour metastasis. Our data further suggest that HCK reciprocally regulates the phosphorylation of TfR‐1 at S24, establishing a positive feedback loop. Phosphorylation of HCK at Y411 stabilises the small N‐terminal loop within its SH1 domain, which consists of five β‐strands and an α‐helix responsible for ATP binding.^40^, ^41^ This phosphorylation event enhances the stability of the SH1 domain, promoting HCK kinase activity and its ability to modulate cellular signalling. We demonstrated that phosphorylation of HCK Y411 significantly boosts its catalytic activity, facilitating the phosphorylation of both TfR‐1 and STAT3. Conversely, mutation of Y411 disrupts the spatial proximity or stable interaction between TfR‐1 and HCK, thereby impairing efficiently phosphorylation of TfR‐1 at Ser24. This sequential phosphorylation cascade potentiates downstream signalling, ultimately driving breast cancer metastasis. These results unequivocally establish the essential role of HCK in breast cancer metastasis.

Furthermore, our study reveals that the stability of HCK is regulated by USP32‐mediated deubiquitylation. Our observation showed that TfR‐1 overexpression or activation concurrently enhanced HCK phosphorylation and elevated its basal protein expression. It has been reported that the mammalian proto‐oncogene Cbl at the plasma membrane induces HCK degradation through the ubiquitin/proteasome dependent manner.^42^ In this study, we illuminated for the first time that USP32 binds to and stabilise HCK protein in breast cancer cells. USP32 exhibits diverse, context‐dependent roles in tumour progression, with its oncogenic functions largely dictated by its specific substrates within different cancers.^43^, ^44^ Although increased USP32 copy number has been linked to breast cancer metastasis,^45^ the mechanisms underlying its pro‐metastasis activity remain unclear. Our study demonstrated that TfR‐1‐induced USP32 activation promotes HCK deubiquitylation and subsequent accumulation. More importantly, we identified S1364 phosphorylation on USP32 as a critical regulatory site that governs its deubiquitinating enzyme activity and a dedicated docking site for TfR‐1. TfR‐1 and USP32 act in coordination to promote HCK stability and activity, triggering a panel of signalling including STAT3 and MMP9 that are critically involved in cell migration and intercellular crosstalk. Collectively, our findings demonstrate the regulatory mechanism for this novel interaction and its dynamic process, exemplifying the diversity of spatiotemporal regulation through reversible ubiquitylation.

Overall, our study reveals a potent pro‐metastatic role of TfR‐1 in breast cancer, driven primarily through the activation of HCK. More importantly, we elucidate that the HCK‒STAT3‒MMP9 axis executes TfR‐1's function in regulating breast cancer metastasis. Our molecular model uncovers a novel function of TfR‐1 extending beyond iron homeostasis regulation, emphasising the potential significance of developing TfR‐1‐targeted strategies. Targeting the TfR‐1‒HCK‒STAT3 pathway rather than iron absorption may effectively suppress breast cancer malignancy without disrupting systemic iron metabolism, representing a safer anti‐tumour approach. However, future therapeutic development should also carefully evaluate the potential impact on iron metabolism and the systemic toxicity that may arise from its disruption.

## 4 METHODS

### 4.1 Cell culture

MCF‐7, MDA‐MB‐231 and 4T‐1 cells were purchased from the American Type Culture Collection (ATCC). MCF‐7 cells were cultured in Minimum Essential Medium, MDA‐MB‐231 cells were cultured in Dulbecco's Modified Eagle Medium (DMEM), and 4T‐1 cells were cultured in RPMI‐1640 medium. HEK293T human embryonic kidney cells were obtained from ATCC and cultured in DMEM. MDA‐MB‐231/4175 cells were a generous gift from Professor Joyce Slingerland (Georgetown University), and were cultured in DMEM. All media were supplemented with 10% foetal bovine serum (FBS). Stable cell lines were established through lentiviral infection and grown in medium containing appropriate antibiotics (1‒5 µg/mL puromycin). All cells were tested negative for mycoplasma contamination, and identified by STR sequence analysis.

### 4.2 Animal procedures

All animal experiments were approved by the Animal Care and Use Committee of Shanghai Institute of Materia Medica (approval nos. 2017‐04‐DJ‐26 and 2019‐05‐DJ‐48), and the experiments were performed following the ethical guidelines for animal care. Female mice (4‒6 weeks) were maintained under specific pathogen‐free conditions with sufficient food and water.

To track tumour growth in vivo, MDA‐MB‐231/4175 mock and TfR‐1‐overexpressing (#1 and #2) cells were subcutaneously injected into the BALB/cA nude mice (*n* = 6 each group). For 4T‐1 cell‐inoculated tumour model, female BALB/cA mice were injected with 4T‐1 mock, or TfR‐1‐overexpressing (#1 and #2) cells into the mammary fat pad (*n* = 6 each group). Tumour volume measurements were calculated using the formula: *V* = 1/2 × length × width^2^.

To evaluate tumour metastasis in vivo, mock, TfR‐1‐overexpressing (#1 and #2), or TfR‐1‐knockdown MDA‐MB‐231/4175 cells were injected intravenously into female BALB/cA nude mice to mimic lung metastasis. Metastasis incidence was assessed 10 days after injection. In another batch of study, lung metastasis was observed on 19th day after injection by in vivo bioluminescence imaging using the IVIS Lumina II (PerkinElmer). And lung tissues were surgically harvested and further analysed by haematoxylin and eosin (H&E) and immunohistochemical staining.

For the orthotopic metastasis model, 4T‐1 mock or TfR‐1‐overexpressing (#1 and #2) cells were injected into the mammary fat pad of mice. Eighteen days after injection, mice were euthanised and the number of lung nodules were assessed.

### 4.3 Chemicals and reagents

The functional‐grade anti‐TfR‐1 monoclonal antibody OKT9 (#16‐0719‐85) was purchased from eBioscience. RK‐20449 (#HY‐15764) and CHX (#HY‐12320) were obtained from MedChemExpress. BP‐1‐102 (#S7769), Sulphorhodamine B (SRB) (#S5976) and MG132 (#S2619) were purchased from Selleck. Tris‒HCl (#ST774), NaCl (#ST1840), MgCl_2_ (#Y001529), dithiothreitol (DTT) (#ST040), EDTA (#ST066) and NP‐40 (#P0013F) were purchased from Beyotime Biotechnology. Glycerol (#G0854) was obtained from Sangon Biotech.

Primary monoclonal antibodies were obtained from the following sources: TfR‐1 (#13113), USP32 (#47247), HCK (#14643), STAT3 (#9139), phospho‐STAT3 (Tyr705) (#4113), Flag‐Tag (#14793), HA‐Tag (#3724), Myc‐Tag (#2276) and GAPDH (#2118) were all purchased from Cell Signalling Technology; phospho‐TfR‐1 (Ser24, #ab61021) and phospho‐HCK (Tyr411, #ab61055) from Abcam; and pan Phospho‐Serine/Threonine (#AP1475), pan Phospho‐Tyrosine Rabbit mAb (#AP1316) and MMP9 (#A0289) from Abclonal.

Secondary antibodies included Alexa Fluor 568‐conjugated antibodies (Life Technologies), Alexa Fluor 488 goat anti‐mouse IgG (H+L) and Alexa Fluor 594 goat anti‐rabbit IgG (H+L), all from Life Technologies. For WB, the secondary antibodies Peroxidase AffiniPure Goat Anti‐Rabbit IgG (H+L) (#111‐035‐003) and Peroxidase AffiniPure Goat Anti‐Mouse IgG (H+L) (#115‐035‐003) were purchased from Jackson ImmunoResearch.

### 4.4 Cell proliferation assay

Mock and TfR‐1‐overexpressed cells were seeded into 96‐well plates (1500‒2000 cells/well) using three replicate wells for each group. The cells were incubated in cell culture CO_2_ incubator for 96–144 h, and the number of cells was counted every 24 h by SRB assay. Proliferation curves were plotted using GraphPad Prism 6 software (www.graphpad.com).

### 4.5 Transwell assay

For cell migration assays, tumour cells were seeded in the upper compartment of a Transwell chambers (Corning, #3464) and were cultured with FBS‐free media, while the lower compartment was filled with complete media containing corresponding compounds. After 24 h, cells were fixed with 4% paraformaldehyde (Beyotime Biotechnology, #P0099). The non‐migrated cells in the upper chamber were wiped off with cotton swabs and the filter was stained for 10 min with .1% crystal violet (Beyotime Biotechnology, #Y268090) in 20% methanol and rinsed with phosphate‐buffered saline (PBS). ImageJ (National Institutes of Health) was used to quantify the cells that had migrated across the filters.

### 4.6 Plasmids and transfection

A pCMV‐Flag‐USP32 WT construct was kindly provided by KyungAh Kim (Samsung Advanced Institute of Technology). A Flag‐USP32 S1364A mutant was generated via site‐directed mutagenesis according to the manufacturer's instructions (Takara Bio). The pLenti‐TFRC plasmid construction as follows: the *TFRC* fragment was cloned from genome DNA and inserted into the pLenti‐EF1a‐P2A‐Puro‐CMV plasmid with EcoRI site.

HA‐Ubiquitin was bought from Addgene (cat no. 18712). Plasmids pcDNA3.2/DEST/hTfR‐HA and pHAGE‐USP32‐EPHA1 both from addgene were used as templates to construct plasmids below: pcDNA3.1/myc‐HisA(+)‐TfR‐1, cloning site: EcoRI/XbaI; pcDNA3.1/Flag‐HA (+)‐USP32, cloning site: BamHI/XhoI; pGEX‐6p‐1‐TfR‐1, cloning site: BamHI/XhoI; pGEX‐6P‐1‐USP32, cloning site: BamHI/XhoI. For the truncated TfR‐1 constructs, TfR‐1‐NT (N‐terminus), ‐PA (protease‐associated domain), ‐M28 (peptidase family M28 domain) and ‐DD (dimerisation domain) were separately cloned into pcDNA3.1/myc‐HisA (+) vectors. For the truncated USP32 constructs, USP32‐EF‐hand1 (21‒126 aa), EF‐hand2 (168‒263 aa), EF‐hand3 (204‒299 aa), DUSP (369‒585 aa), UBL (586‒733 aa) and UCH (734‒1601 aa) were separately inserted into pcDNA3.1/Flag‐HA (+) and pGEX‐6P‐1 vectors. The PLV‐NEO‐CMV‐HCK plasmid was synthesised by Tsingke Technology. The variants of TfR‐1 (Y20F and S24I), USP32 (S1364A) and HCK (Y411F) were cloned with Mutagenesis Kit (TOYOBO, SMK‐101) and inserted into pcDNA3.1 or PLV backbone. All plasmids were sequenced and confirmed for accuracy.

Plasmid and siRNA transfections were performed using Lipofectamine 2000 (Thermo Fisher Scientific) and Lipofectamine RNAiMAX (Thermo Fisher Scientific), respectively, following the manufacturers' protocols. Lentiviral particles were generated in HEK293T cells using a standard packaging protocol, and subsequently employed to establish stable cell lines. The siRNA sequences used in this study are listed in Table S2.

### 4.7 CRISPR/cas9 knockdown of TfR‐1 and HCK

The guide RNA‐targeting TfR‐1 plasmid pU6‐sgCD71‐2 (#46918) and the CRISPR universal negative control plasmid lentiCRISPR v2‐Puro (#98290) were obtained from Addgene. The sgRNA‐mediated HCK knockout (sgHCK) construct was generated by inserting the HCK‐targeting sequence into the lentiCRISPR v2‐Puro vector, with technical support from Beijing Qingke Biotechnology. Cells were transfected with the indicated plasmids and selected in complete growth medium containing puromycin (1 µg/mL, Thermo Fisher Scientific, #A1113802) for 2–3 weeks. Single‐cell clones were isolated using 96‐well plates. Knockdown of TfR‐1 and HCK was confirmed by WB and PCR genotyping to verify exon‐targeted insertion of the Puro/RFP cassette. For maintenance of stable cell lines, puromycin was used at .2 µg/mL.

### 4.8 Human phospho‐kinase arrays

The human phospho‐kinase array kit (#ARY003B) was purchased from R&D Systems and human RTK phosphorylation array (AAH‐PRTK‐1‐2) was purchased from RayBiotech. Array screening was performed following the manufacturers’ instructions. Briefly, cell lysates were incubated with the array membranes. After washing, the membranes were incubated with biotinylated antibody cocktail. The amounts of phosphokinase were assessed with streptavidin conjugated to horseradish peroxidase (HRP), followed by chemiluminescence detection. A GS‐800 Calibrated Densitometer (Bio‐Rad Laboratories) was used to quantify the density of each dot against the average of the internal controls on the membrane as indicated in the protocol.

### 4.9 Real‐time quantitative PCR analysis

Total RNA was isolated using RNA easy Minikit (Qiagen, #74106), and reverse transcript with Superscript III kit (Invitrogen, #18080051). Real‐time quantitative PCR (RT‐qPCR) was carried out according to the instructions for SYBR Green PCR master mix (Bio‐Rad, #6252) by V7 Real‐Time PCR system (Applied Biosystems). Relative gene mRNA expression was quantified using the 2^−ΔΔCT^ method. Primers were synthesised by Sangon Biotech and the sequences are listed in Table S1. All mRNA expression was normalised to *GAPDH*.

### 4.10 RNA‐seq analysis

Total RNA was isolated from MDA‐MB‐231/4175 Mock and TfR‐1‐overexpressing cells. RNA‐seq was carried out on the Illumina HiSeq platform using the standard paired‐end protocol. Mapping of RNA‐seq reads was performed with HISAT2 (http://ccb.jhu.edu/software/hisat2/index.shtml/) and the human RefSeq gene model (GRCh38.p10) and RSEM (http://deweylab.github.io/RSEM/) software were used to quantify gene expression. The differentially expressed gene analyses were performed using DESeq2, GO enrichment analysis was performed with GOATOOLS (https://pypi.org/project/goatools/).

### 4.11 Co‐IP and WB analysis

For immunoprecipitation, cells were harvested into 1.5‐mL microcentrifuge tubes after the indicated treatments. Cells were lysed in 1 mL NP‐40 lysis buffer (50 mM Tris‒HCl, pH 7.5; 150 mM NaCl; 1% NP‐40; 10% glycerol; 1 mM EDTA) supplemented with protease and phosphatase inhibitor cocktails, incubated on ice for 20 min, and centrifuged at 13 000 × *g* for 15 min at 4°C. An aliquot of 50 µL supernatant was reserved as the input. The remaining lysate was incubated with the indicated primary antibodies or IgG control overnight at 4°C, followed by incubation with Protein A/G agarose beads (Santa Cruz Biotechnology, #sc‐2003) for 4 h at 4°C the next day. Beads were washed five times with NP‐40 lysis buffer, resuspended in 50 µL 1× SDS sample buffer, and boiled for 5 min. Eluted proteins were then analysed by WB. For WB analysis, cells were collected and lysed with SDS lysis buffer. After boiling, the samples were resolved by SDS‒PAGE gel and immunoblotted with standard protocols. Immunoblots were quantified using the ImageJ software (https://imagej.nih.gov/ij/), which measured the integrated density of bands corrected for background.

### 4.12 Deubiquitination assay

For in vitro debuiquitination assay, HEK293T cells co‐expressing HA‐ubiquitin and Flag‐tagged HCK were treated with 10 µM MG132 overnight. Polyubiquitinated HCK was purified using Flag‐antibody‐conjugated beads. Then, the same amounts of beads were incubated with or without 10 ng/µL recombinant full length USP32 WT protein or the S1364A mutant in the deubiquitination reaction buffer (50 mM Tris‒HCl pH 7.4, 150 mM NaCl, 5 mM MgCl_2_ and 10 mM DTT) at 37°C for 1 h. The reaction was terminated by the addition of SDS loading buffer. The poly‐ubiquitination status of HCK was analysed by anti‐HA blotting.

### 4.13 In vitro kinase assay

HEK293T cells were transfected with HA‐TfR‐1, lysed, and immunoprecipitated using anti‐HA agarose beads. One microgram of the agarose‐purified HA‐TfR‐1 protein was incubated with purified human HCK protein in kinase reaction buffer (50 mM Tris‒HCl, pH 7.5; 1 mM MgCl_2_; 2 mM DTT; 1 mM EGTA) containing 200 µM ATP for 30 min at 30°C. Subsequently, phosphorylation of HCK was assessed by immunoblotting.

### 4.14 Immunofluorescence staining

Cells were plated on glass coverslips and treated with indicated inhibitors for 48 h. Cells were fixed with 4% paraformaldehyde after washing and applied with .05% Triton X‐100 for permeabilisation. After blocking with 10% bovine serum albumin solution, cells were incubated with indicated antibody (dilution ratio 1:100) for overnight. Alexa Fluor 633 goat anti‐rabbit antibody (#A‐21071) and Alexa Fluor 488 goat anti‐mouse antibody (#A‐11001) (dilution ratio 1:500; Molecular Probes) were used as secondary antibody. Cell nuclei were stained with nuclear binding 4′,6‐diamidino‐2‐phenylindole (DAPI). Fluorescence analyses were performed with an Olympus Fluor view 1000 confocal microscope.

### 4.15 IHC staining of breast cancer tissue microarray

All human breast cancer tissue microarray samples used in this study were provided by ZuoCheng Biotechnology, which has completed standardized ethical review and acquired written informed consent from all enrolled patients before preparing commercial tissue chips. All analyses involving human tissue specimens strictly comply with the tenets of the Declaration of Helsinki. The collection, processing, immunohistochemical staining, and initial assessment of TfR‐1 expression in a human breast cancer tissue microarray were performed by ZuoCheng Biotechnology. A total of 140 breast cancer tissue samples were obtained from patients who underwent surgical resection as their primary treatment. TfR‐1 expression was semi‐quantitatively evaluated using the *H*‐score method, which integrates staining intensity (0 = no staining, 1 = weak, 2 = moderate, 3 = strong) and the percentage of positive cells. The final *H*‐score was calculated independently, as the sum of the percentage of cells at each intensity level multiplied by the corresponding intensity score, resulting in a total score ranging from 0 to 300.

### 4.16 Enzyme‐linked immunosorbent assay

MMP9 in cell culture supernatants was measured using the MMP9 enzyme‐linked immunosorbent assay (ELISA) kit (Abclonal, #RK00217). Samples and standards were incubated with a biotin‐labelled MMP9 antibody, and followed by detection using HRP‐conjugated streptavidin. 3,3′,5,5′‐Tetramethylbenzidine substrate was added, and absorbance at 450 nm was measured to determine MMP9 concentrations based on the standard curve.

### 4.17 Bioinformatics analysis

Human breast cancer gene expression data from non‐metastatic and metastatic cases (GSE20685, GSE14020, GSE147007 and GSE46141) were obtained from the GEO database. After batch‐effect correction, differential expression analysis was performed using the ‘limma’ package in RStudio.

### 4.18 Statistical analyses

Statistical analyses were performed. Means, standard deviation (SD) and standard error of the mean (SEM) were analysed using GraphPad Prism software (version 5.0). Two‐tailed Student's *t*‐test, two‐way ANOVA, or one‐way ANOVA with Dunnett's multiple comparisons test were used to compare the statistical difference between indicated groups. Statistical significance was accepted for *p* values of <.05. Correlation was studied using the Spearman test.

## AUTHOR CONTRIBUTIONS

Yi Chen, Yafang Wang and Qing Zhao designed the research. Qing Zhao and Yafang Wang performed the research and analysed the data. Qing Zhao contributed to bioinformatics analysis. Pengfei Wang, Yaqi Ding and Rong Wang contributed to the conduction of in vitro experiments. Yanyan Shen and Biyu Yang contributed to the in vivo studies and provided technical support during the experiments. Qing Zhao and Yafang Wang wrote the manuscript which was revised by Yi Chen, Jian Ding and Yanfen Fang. Yi Chen and Jian Ding provided funding and resources. All the authors discussed the results and approved the final manuscript.

## CONFLICT OF INTEREST STATEMENT

The authors declare they have no conflicts of interest.

## Supporting information



Supporting Information

Supporting Information

## Data Availability

All data reported in this study are available from the lead contact Yi Chen (ychen@simm.ac.cn), upon reasonable request. The source code generated in this study is publicly available on GitHub at: https://github.com/qingzhao1226/ML‐based‐TfR1‐TMA‐prognostic‐analysis. The RNA‐seq data generated in this study can be accessed from the Gene Expression Omnibus (GEO) database under the accession number GSE318333.
