# Supplementary material for: Non‐canonical function of transferrin receptor‐1 promotes breast cancer metastasis by activating HCK‒STAT3‒MMP9 signalling
Source: Clin Transl Med. 2026 Jul 12;16(7):e70731. doi: 10.1002/ctm2.70731 (PMC13357684; doi:10.1002/ctm2.70731)
Supplement: Supplementary file 1 — Supporting Information [file CTM2-16-e70731-s001.docx]

**Supplementary information**

**Non-canonical function of transferrin receptor-1 promotes breast cancer metastasis by** **activating HCK-STAT3-MMP9 signaling**

Qing Zhao^1,3#^, Yafang Wang^2,5#*^, Pengfei Wang^2^, Yaqi Ding^2^, Rong Wang^2^, Yanyan Shen^3^, Biyu Yang^3^, Yanfen Fang^3^, Jian Ding^1,3,4*^, Yi Chen^2*^

**This file inclues:**

- Figure S1 TfR-1 overexpression accelerates breast tumor growth and metastasis in preclinical models
- Figure S2 TfR-1 activates the HCK-STAT3 signaling axis
- Figure S3 TfR-1 promotes HCK phosphorylation via its N-terminus
- Figure S4 TfR-1 stabilizes HCK protein through deubiquitinase USP32
- Figure S5 MMP9 is a central downstream executor in TfR-1-promoted breast cancer cells mobility


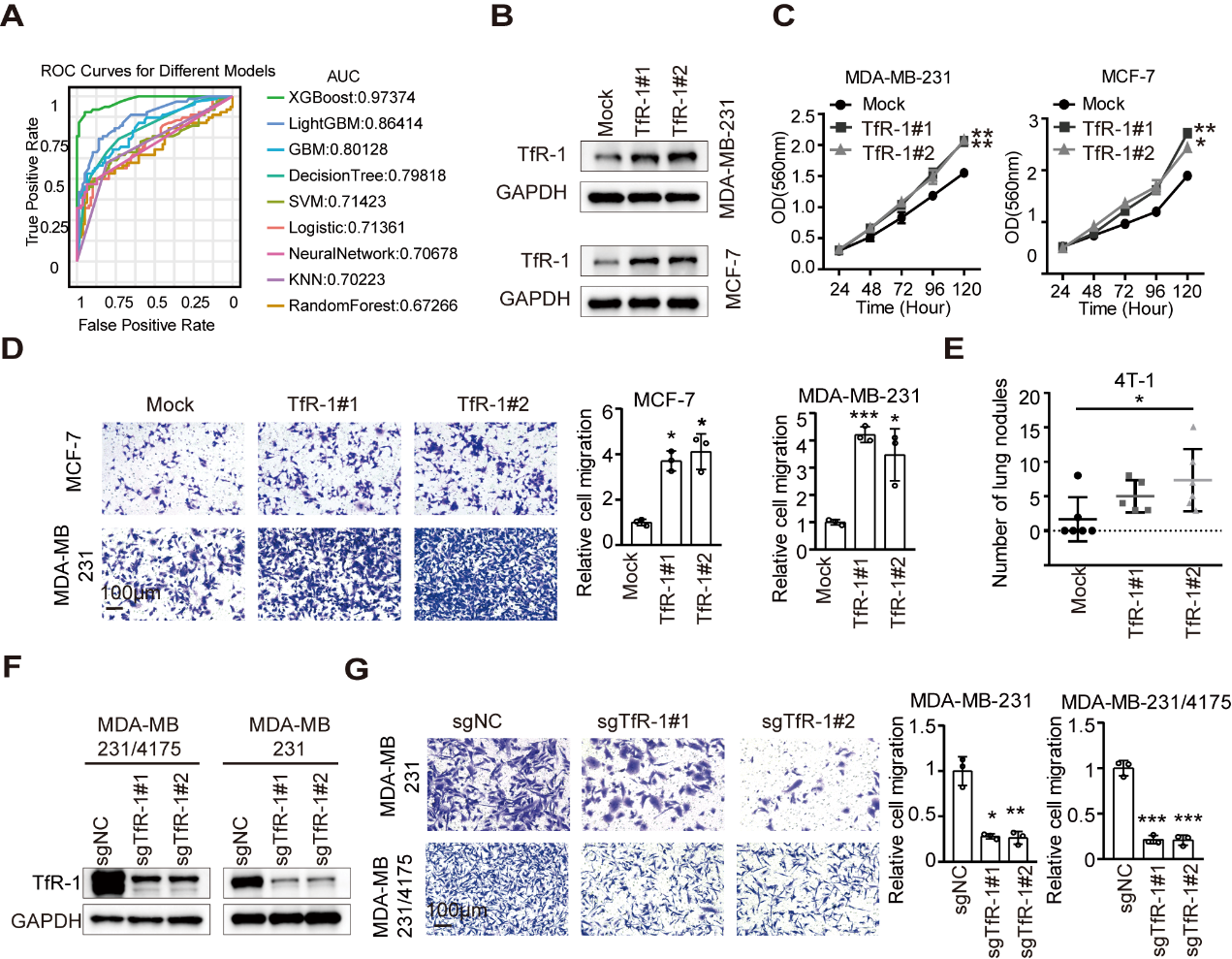


**Figure S1 TfR-1 overexpression accelerates breast tumor growth and metastasis in preclinical models**

(A) The optimal cutoff value distinguishing high and low TfR-1 expression groups was determined using multiple machine learning models and evaluated by receiver operating characteristic (ROC) curve analysis. (B) WB analysis confirmed TfR-1 overexpression in MDA-MB-231 and MCF-7 cell lines. (C) Growth curve of mock and TfR-1 overexpressed MDA-MB-231 and MCF-7 cells. (D) Transwell migration analysis revealed that TfR-1 overexpression enhanced the migratory ability of MDA-MB-231 and MCF-7 cells. (E) The number of lung metastatic nodules was assessed 18 days after implantation of mock- and TfR-1-overexpressing 4T-1 tumor cells (n = 6/group). (F) TfR-1 knockout in MDA-MB-231/4175 and MDA-MB-231 cell lines was validated by WB analysis. (G) Transwell migration analysis demonstrated that knockout of TfR-1 significantly reduced the migratory capacity of both MDA-MB-231/4175 and MDA-MB-231 cells. All data are presented as mean values ± SEM. Statistical significance was assessed using an unpaired, two-tailed Student's t-test, **p* < 0.05, ***p* < 0.01, ****p* < 0.001.


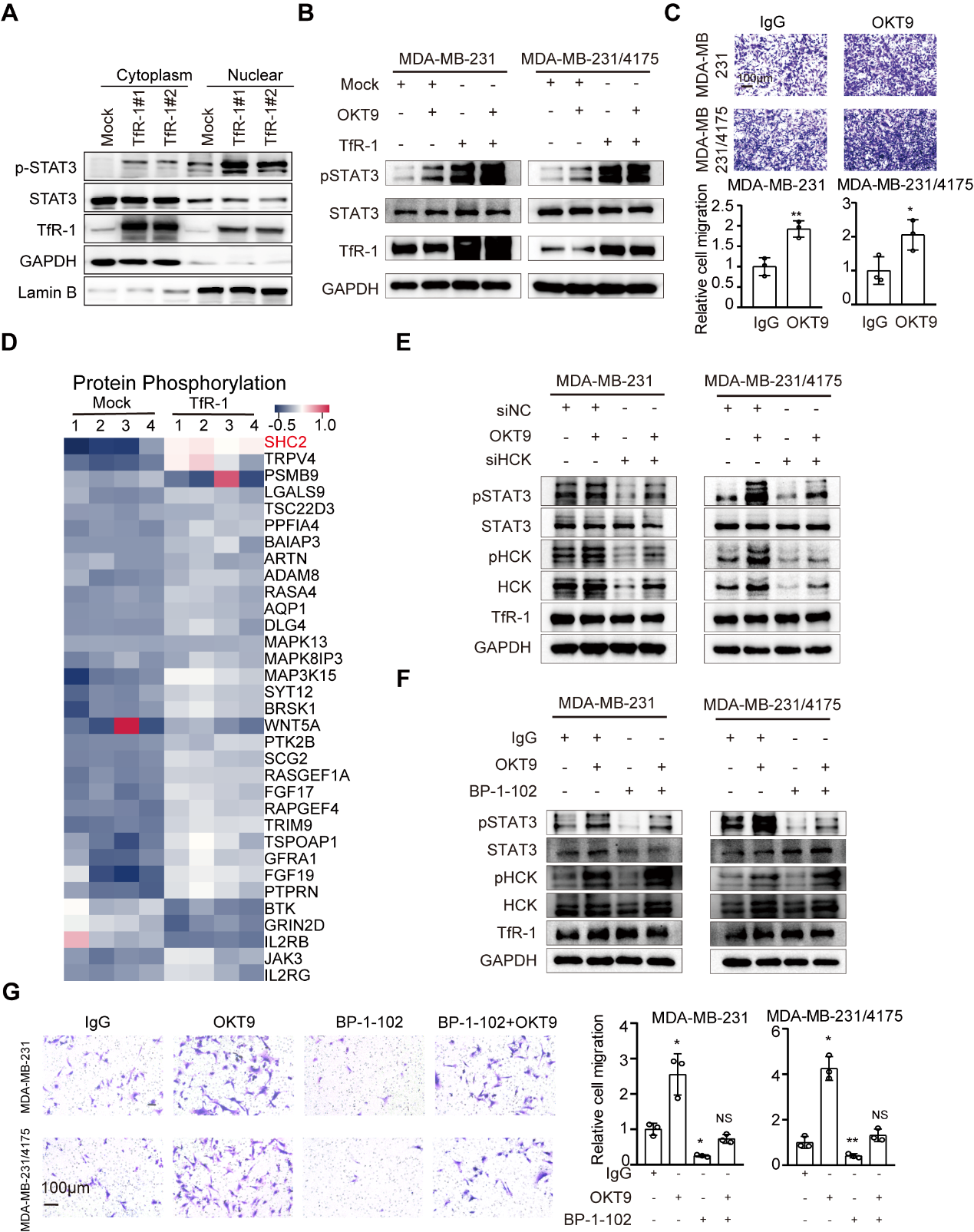


**Figure S2** **TfR-1 activates the HCK-STAT3 signaling axis**

(A) Following nuclear-cytoplasmic fractionation, protein expression levels were analyzed by WB using specific antibodies. (B) After treatment with OKT9, the level of STAT3 and pSTAT3 in breast cancer cells transfected with mock or TfR-1 were detected using WB analysis. (C) Cells migration ability were examined after treatment with OKT9. (D) GO enrichment analysis of RNA-seq data comparing mock-transfected and TfR-1-overexpressing MDA-MB-231/4175 cells. The heatmap shows differential expression of related genes. (E) Indicated proteins expression was analyzed in HCK-depleted MDA-MB-231 and MDA-MB-231/4175 cells after treatment with or without OKT9. Reversal effect on OKT9-mediated signaling pathway (F) and cell migration ability (G) by STAT3 inhibitor BP-1-102 (10 μM, 24h). Statistical significance was assessed using an unpaired, two-tailed Student's t-test, **p* < 0.05, ***p* < 0.01, ****p* < 0.001.


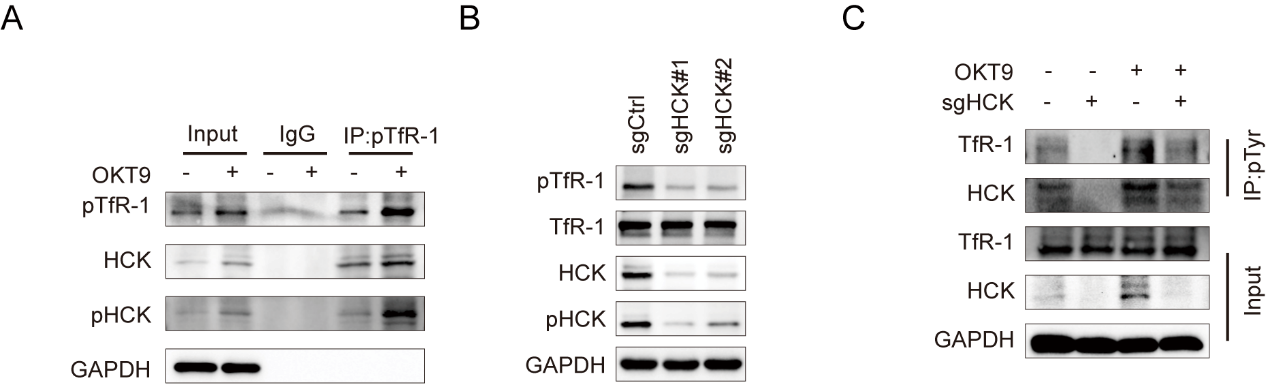


**Figure S3 TfR-1 promotes HCK phosphorylation via its N-terminus**

(A) MDA-MB-231/4175 cell lysates were subjected to immunoprecipitation with pTfR-1 antibody, and analyzed by immunoblotting for HCK and pHCK. (B) TfR-1 and phosphorylation TfR-1 were analyzed in HCK-depletion MDA-MB-231/4175 cells. (C) The global tyrosine phosphorylation of TfR-1 and HCK were evaluated in HCK-knockout MDA-MB-231/4175 cells treated with OKT9.


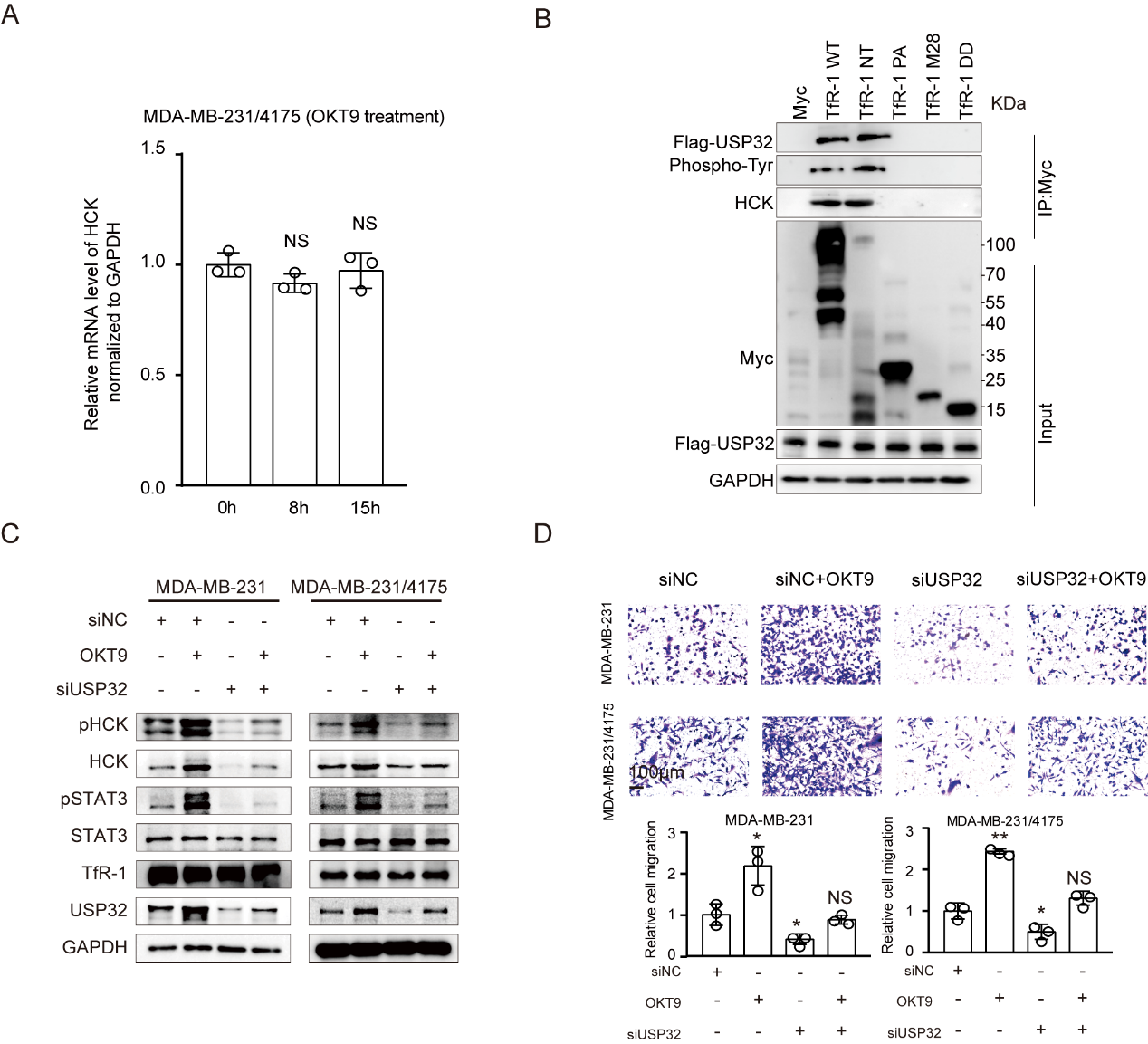


**Figure S4 TfR-1 stabilizes HCK protein through deubiquitinase USP32**

(A)The mRNA levels of HCK in MDA-MB-231/4175 cells treated with OKT9 for indicated time were analyzed by RT-qPCR. (B) Myc-TfR-1 wild type, four truncated mutants, empty vector with Myc-tag and Flag-USP32 were expressed in HEK293T cells, respectively. Whole cell lysates were IPs of Myc-tag, then detected by WB with anti-USP32, anti-HCK, anti-Flag and anti-phospho-Tyr antibodies. In breast cancer cells lacking USP32, the effect of OKT9 on indicated proteins (C) and cells migration (D) were assessed. Statistical significance was assessed using an unpaired, two-tailed Student's t-test, **p* < 0.05, ***p* < 0.01, ****p* < 0.001.


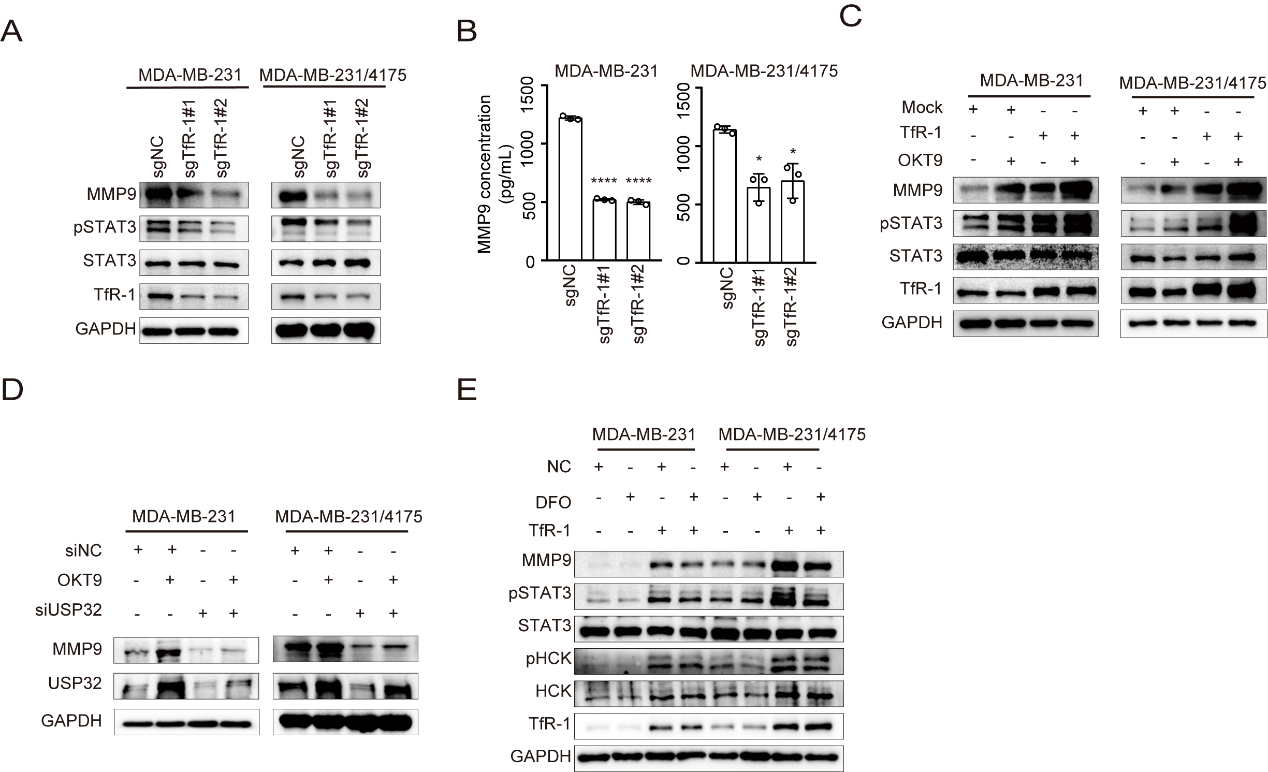


**Figure S5 MMP9 is a central downstream executor in TfR-1-promoted breast cancer cells mobility**

(A) WB analysis of total STAT3, pSTAT3, and MMP9 in MDA-MB-231 and MDA-MB-231/4175 control or TfR-1-knockdown cells. (B) ELISA measurement of secreted MMP9 in TfR-1-depleted MDA-MB-231 and MDA-MB-231/4175 cells. (C) WB analysis of MMP9, pSTAT3, STAT3, and TfR-1 in TfR-1-overexpressing MDA-MB-231 and MDA-MB-231/4175 cells after treatment with or without OKT9. (D) WB analysis of MMP9 and USP32 in USP32-depleted MDA-MB-231 and MDA-MB-231/4175 cells after treatment with or without OKT9. (E) Expression of proteins in the HCK-STAT3 signaling pathway was evaluated in NC and TfR1-overexpressing groups of MDA-MB-231 and MDA-MB-231/4175 cells, with or without treatment with the iron chelator DFO. Data are shown as mean±SD from at least three independent experiments. Statistical significance was determined by unpaired, two-tailed Student's t-test (**p* < 0.05, ***p* < 0.01, ****p* < 0.001, *****p* < 0.0001) versus respective controls.
