# Supplementary material for: Non‐canonical function of transferrin receptor‐1 promotes breast cancer metastasis by activating HCK‒STAT3‒MMP9 signalling
Source: Clin Transl Med. 2026 Jul 12;16(7):e70731. doi: 10.1002/ctm2.70731 (PMC13357684; doi:10.1002/ctm2.70731)
Supplement: Supplementary file 2 — Supporting Information [file CTM2-16-e70731-s002.docx]

**Supplementary information**

**Non-canonical function of transferrin receptor-1 promotes breast cancer metastasis by** **activating HCK-STAT3-MMP9 signaling**

Qing Zhao^1,3#^, Yafang Wang^2,5#*^, Pengfei Wang^2^, Yaqi Ding^2^, Rong Wang^2^, Yanyan Shen^3^, Biyu Yang^3^, Yanfen Fang^3^, Jian Ding^1,3,4*^, Yi Chen^2*^

**This file inclues:**

- Table S1 Sequences in RT-qPCR analysis
- Table S2 The target sequences of siRNAs

**Table S1 Sequences in RT-qPCR analysis**

| Sequences in RT-qPCR analysis | | |
| --- | --- | --- |
| Genes | Forward (5’-3’) | Reverse (5’-3’) |
| h*GAPDH* | AATGGGCAGCCGTTAGGAAA | GCGCCCAATACGACCAAATC |
| h*TFRC* | GGACGCGCTAGTGTTCTTCT | CATCTACTTGCCGAGCCAGG |
| h*MMP9* | TGTACCGCTATGGTTACACTCG | GGCAGGGACAGTTGCTTCT |
| hHCK | CAGGATGGGGTGCATGAAGT | CCTCCCTGATTCCTGGTGTG |

**Table S2 Target sequences for siRNAs and sgRNAs**

| Name | Target sequences |
| --- | --- |
| siNC | UUCUCCGAACGUGUCACGUTT |
| siUSP32 #1 | CCAGUAAAGGCUACAUCAU |
| siUSP32 #2 | GCCUCAGUUACGUGAAUAC |
| siHCK #1 | GUCGGAGGCAAUACAUUCUTT |
| siHCK#2 | CACUAAAGGAAGCUACUCUTT |
| siMMP9 #1 | CCACAACAUCACCUAUUGGAUTT |
| siMMP9 #2 | CAGUUUCCAUUCAUCUUCCAATT |
| sgHCK #1 | CACCGGGTTGCCCTGTATGATTACG |
| sgHCK #2 | CACCGATGTATTGCCTCCGACCTGG |
